# Outcomes of Cervical Cancer Treatment Using Total Mesometrial Resection (TMMR) Performed with the Robotic System—A Preliminary Report

**DOI:** 10.3390/jcm14248667

**Published:** 2025-12-07

**Authors:** Marcin Opławski, Krzysztof Mawlichanów, Agnieszka Golec-Cera, Anna Jedrzejczyk, Kazimierz Pitynski, Radovan Pilka

**Affiliations:** 1 Department of Gynecology and Obstetrics, Faculty of Medicine and Health Sciences, Andrzej Frycz Modrzewski Kraków University, 30-705 Cracow, Poland; anna@jedrzejczyk.com.pl; 2 Department of Gynecology, Neo Hospital, 30-437 Cracow, Poland; mawlichanow@gmail.com (K.M.); agagoleccera@gmail.com (A.G.-C.); kazimierz.pitynski@uj.edu.pl (K.P.); radovan.pilka@fnol.cz (R.P.); 3 Department of Gynecology and Obstetrics with Gynecologic Oncology, Ludwik Rydygier Memorial Specialized Hospital, 31-820 Cracow, Poland; 4 Bioethics and Medical Law Department, Andrzej Frycz Modrzewski Kraków University, 30-705 Cracow, Poland; 5 Department of Gynecology and Oncology, Jagiellonian University Medical College, 31-501 Krakow, Poland; 6 Department of Obstetrics and Gynecology, Faculty of Medicine and Dentistry, Palacky University, University Hospital Olomouc, 779 00 Olomouc, Czech Republic

**Keywords:** cervical cancer, total mesometrial resection (TMMR), robotic surgery, da Vinci system, oncologic outcomes

## Abstract

**Background/Objectives:** Cervical cancer remains a major cause of cancer-related morbidity and mortality among women worldwide. The introduction of total mesometrial resection (TMMR), based on the ontogenetic compartment theory, has redefined the concept of surgical radicality in cervical cancer treatment. This study aimed to evaluate the perioperative, histopathological, and early oncologic outcomes of TMMR performed using the da Vinci Xi robotic system in patients with early-stage cervical carcinoma. **Methods:** A pilot, prospective, single-center study was conducted between 2021 and 2023 and included 20 consecutive patients diagnosed with Fédération Internationale de Gynécologie et d’Obstétrique (FIGO) stage IA2–IIA1 cervical carcinoma. All patients underwent robotic surgery: 4 classic radical robotic hysterectomies, 12 radical robotic hysterectomies using the TMMR technique with pelvic lymphadenectomy, and—given the young age of selected patients, fertility considerations, and tumor characteristics—4 radical trachelectomies. Surgical parameters, histopathological data, and 24-month follow-up outcomes were analyzed. Statistical analyses included Spearman’s correlation, Fisher’s exact test, and Mann–Whitney U test, with *p* < 0.05 considered statistically significant. **Results:** All procedures were completed robotically without conversion to laparotomy. The mean operative time was 178 ± 42 min, mean blood loss 112 ± 61 mL, and mean hospital stay 4.2 ± 1.6 days. No intraoperative complications occurred. Minor postoperative complications (Clavien–Dindo grade I–II) were observed in 10% of cases. Negative surgical margins (R0) were achieved in 17 cases, while positive margins (R+) were observed in 4 cases. Lymph node metastases were present in 20.0% of patients, and both lymphovascular space invasion (LVSI) and Vascular Endothelial Growth Factor (VEGF) expression were detected in 33.3%. No significant correlations were found between VEGF expression, LVSI, or nodal status. During the 24-month follow-up period, no local or distant recurrences were documented. **Conclusions:** Robotic TMMR for early-stage cervical cancer is feasible, safe, and provides complete oncologic radicality with low perioperative morbidity. Although these preliminary results are promising, larger multicenter studies are needed to validate long-term oncologic outcomes and to establish standardized protocols for robotic compartment-based surgery.

## 1. Introduction

Cervical cancer remains a major global health burden, ranking among the most common malignancies in women worldwide [1,2]. According to the World Health Organization (WHO), its incidence varies substantially—from approximately 75 per 100,000 women in developing countries to about 10 per 100,000 in high-income nations—reflecting significant disparities in prevention, early detection, and access to care [3]. Persistent infection with high-risk human papillomavirus (HPV) types is recognized as the principal etiological factor [4]. The widespread implementation of cytological and HPV-based screening, together with vaccination programs, has markedly reduced both incidence and mortality in high-income regions [5]. However, in many low- and middle-income countries, cervical cancer is still frequently diagnosed at advanced stages, largely due to limited participation in screening programs [6].

Current international guidelines from the European Society of Gynaecological Oncology (ESGO), European Society for Radiotherapy and Oncology (ESTRO), and European Society of Pathology (ESP), as well as national recommendations from the Polish Society of Gynecologic Oncology (PTGO), endorse surgical treatment for early-stage cervical cancer up to International Federation of Gynecology and Obstetrics (FIGO) stage IIA1, with the exception of FIGO IB2 and IIA2, for which concomitant chemoradiotherapy remains the preferred therapeutic option. In the earliest stage (FIGO IA1), conservative management such as conization or simple hysterectomy is sufficient, whereas radical hysterectomy with pelvic lymphadenectomy is standard for FIGO IA2–IIA1 disease [7,8]. The primary aim of surgery is the complete resection of the tumor with negative margins to ensure oncologic radicality. Advances in imaging—particularly pelvic magnetic resonance imaging (MRI)—have enhanced preoperative staging accuracy and patient selection, replacing earlier methods based primarily on physical examination and urography [9,10].

The introduction of minimally invasive techniques initially transformed the surgical management of cervical cancer [11]. However, the landmark Laparoscopic Approach to Cervical Cancer (LACC) trial, published in 2018, demonstrated significantly worse disease-free and overall survival in patients undergoing minimally invasive radical hysterectomy compared with open surgery. As a result, major professional societies recommended open abdominal radical hysterectomy as the standard approach for early-stage disease [12]. Subsequent analyses, including large multicenter studies such as the Safe Use of Conservative and/or Radical Surgery in Cervical Cancer (SUCCOR) study, have questioned the generalizability of the LACC findings, citing methodological limitations, heterogeneous surgeon experience, and variability in surgical techniques. Although the LACC trial reported inferior survival outcomes following minimally invasive radical hysterectomy, later multicenter investigations—such as RACC, MEMORY, and recent Korean and German cohorts—suggest that these differences primarily reflect disparities in surgical expertise, tumor-handling practices, and adherence to oncologic principles rather than the minimally invasive approach itself. Emerging evidence indicates that when performed by experienced surgeons using compartment-based and nerve-sparing approaches such as total mesometrial resection (TMMR), minimally invasive surgery may achieve oncologic outcomes comparable to open procedures while offering improved preservation of pelvic autonomic function [13,14].

In this context, TMMR—introduced by Höckel and based on ontogenetic compartment theory—has redefined the surgical philosophy of cervical cancer treatment [15]. TMMR emphasizes en bloc removal of the Müllerian compartment to ensure local tumor control while preserving autonomic nerves and pelvic function. The integration of robotic technology, particularly the da Vinci surgical platform, enables unparalleled precision, three-dimensional visualization, and meticulous dissection of mesometrial planes, thereby potentially restoring the benefits of minimally invasive surgery without compromising oncologic safety [16,17].

The present preliminary (pilot) study evaluates the perioperative, histopathological, and early oncologic outcomes of TMMR performed with the da Vinci robotic system in patients with early-stage cervical cancer. The aim is to assess surgical radicality, operative parameters, and short-term oncologic results achieved by specialized surgical teams, thereby contributing contemporary evidence to the ongoing discussion regarding the role of robotic TMMR in modern cervical cancer management.

## 2. Materials and Methods

### 2.1. Study Design

This was a prospective, observational, single-center pilot study designed to evaluate the surgical and pathological outcomes of patients with early-stage cervical cancer treated with total mesometrial resection (TMMR), classic radical robotic hysterectomy, or—when clinically indicated—robotic trachelectomy using the da Vinci Xi system. The study formed part of a European Union–funded project aimed at assessing the safety, feasibility, and oncologic adequacy of compartment-based robotic surgery in gynecologic oncology.

Patient recruitment took place at the Department of Gynecology, Neo Hospital, Cracow, Poland, between 2021 and 2023. Twenty-one patients were initially screened for eligibility; one was excluded due to a nonstandard diagnostic work-up, and a postoperative fatality occurring outside the final cohort prompted an institutional ethical review and suspension of further enrollment. Ultimately, 20 patients met all inclusion criteria and completed the planned 24-month follow-up.

The study protocol was approved by the Bioethics Committee of Andrzej Frycz Modrzewski Krakow University, Poland (approval no. KBKA/47/0/2020, issued 9 December 2020), and conducted in accordance with the Declaration of Helsinki. All participants provided written informed consent for both the surgical procedure and anonymized data analysis.

### 2.2. Participants

**Figure 1:**
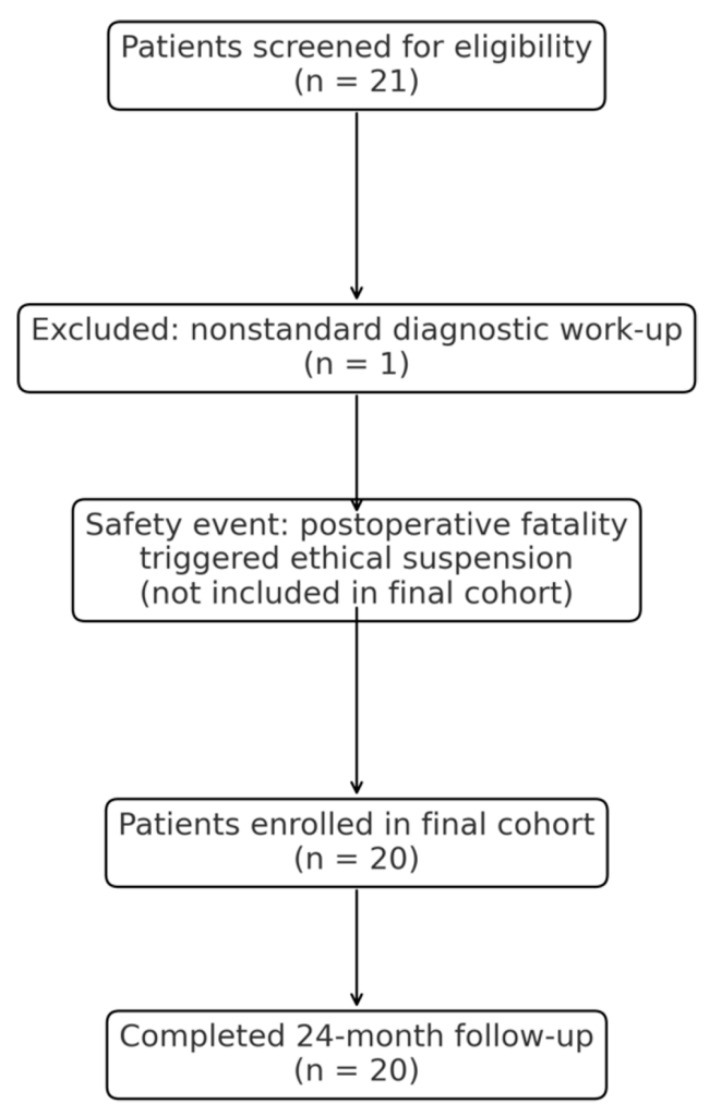
CONSORT-style flow diagram of patient screening, exclusions, safety-related suspension, and final study cohort.

Of the 21 patients initially screened for eligibility, one was excluded because the diagnostic work-up relied solely on expert ultrasonography rather than the full standardized clinical and imaging protocol required for enrollment. In addition, a postoperative intra-abdominal hemorrhage resulting in a fatality occurred outside the final cohort and triggered an institutional ethical review, which led to the suspension of further recruitment. Consequently, the final study population consisted of 20 patients who met all inclusion criteria and completed the 24-month follow-up. A CONSORT-style flow diagram summarizing patient screening, exclusions, the safety-related suspension, and the final study cohort is presented in Figure 1. All patients were diagnosed with cervical carcinoma at FIGO stages IA2 to IIA1 and qualified for primary surgical treatment after comprehensive clinical and imaging evaluation. Eligible participants were adults aged 18 years or older with histologically confirmed disease, an ECOG performance status of 0–2, and suitability for robotic surgery using the da Vinci system. A complete diagnostic work-up—including pelvic examination, colposcopy, pelvic MRI, and biopsy or conization—was required, and distant metastases were excluded on imaging. Patients were excluded if they had disease beyond FIGO stage IIA1, recurrent or locally advanced cancer, prior pelvic radiotherapy or chemotherapy, significant comorbidities contraindicating general anesthesia, incomplete or nonstandard diagnostic evaluation, or pregnancy.

Baseline demographic and clinical characteristics—including age, body mass index (BMI), parity, histological subtype, and tumor size assessed on MRI—were recorded for all participants. Most tumors were classified as squamous cell carcinoma, with adenocarcinoma comprising the remaining cases. The mean age of the cohort was 45.67 ± 9.31 years, and the mean BMI was 25.18 ± 5.18 kg/m^2^. The mean body weight and height were 70.90 ± 16.59 kg and 167.19 ± 6.11 cm, respectively. All patients were in good general condition (ECOG 0–2). All underwent preoperative colposcopy and pelvic MRI for staging, and diagnostic conization was performed in eight cases. HPV infection was confirmed in four patients and HSV infection in two. The mean tumor size on MRI was 1.4 ± 0.7 cm, with all lesions localized within the cervix uteri (Table 1).

### 2.3. Preoperative Evaluation

All patients underwent standardized diagnostic and staging procedures. A pelvic examination, colposcopy, and histopathological verification of the cervical lesion were performed prior to surgery. Magnetic resonance imaging (MRI) of the pelvis served as the primary imaging modality to assess tumor size, stromal invasion, and parametrial involvement. When necessary, transvaginal or transrectal ultrasound performed by experienced sonographers was used to complement the MRI findings.

To exclude distant metastases, either computed tomography (CT) or chest X-ray was obtained. In cases with suspected bladder or rectal infiltration, cystoscopy or rectoscopy was performed to allow direct visualization and biopsy. Preoperative laboratory evaluation included a complete blood count, biochemical panel, coagulation profile, and HPV-DNA testing.

### 2.4. Surgical Technique

All surgeries were performed using the da Vinci Xi robotic platform by a certified gynecologic oncologist together with an experienced, certified assistant surgeon, both specifically trained in robotic TMMR. Patients were positioned in low lithotomy with a steep Trendelenburg tilt. Four robotic trocars and one assistant port were placed. After docking, the uterus and adnexa were mobilized according to the ontogenetic compartment theory described by Höckel, enabling precise excision of the Müllerian compartment, including the mesometrium, mesosalpinx, and paracervical and paravaginal tissues.

The ureters were dissected and preserved under direct three-dimensional visualization, allowing meticulous nerve-sparing whenever oncologically appropriate. Pelvic lymphadenectomy included the obturator, external iliac, and internal iliac nodal basins. Para-aortic lymphadenectomy was performed in four patients (19.0%) due to intraoperative or radiological suspicion of nodal involvement. The median number of pelvic lymph nodes retrieved was 18 (range: 12–27). The vagina was transected approximately 1.5–2 cm below the tumor margin. The specimen was extracted transvaginally using a protective endoscopic bag with a MyTube^®^ vacuum retractor (AMI—Agency for Medical Innovations GmbH, Feldkirch, Austria), and the vaginal vault was closed robotically.

All patients received perioperative antibiotic prophylaxis and thromboprophylaxis. Standardized postoperative care included early mobilization, gradual reintroduction of oral intake, and bladder catheter removal between postoperative days 12 and 18 following successful voiding trials. The mean operative time was 178 ± 42 min, with an estimated blood loss of 112 ± 61 mL. No intraoperative complications occurred, and the mean hospital stay was 3 days.

**Figure 2:**
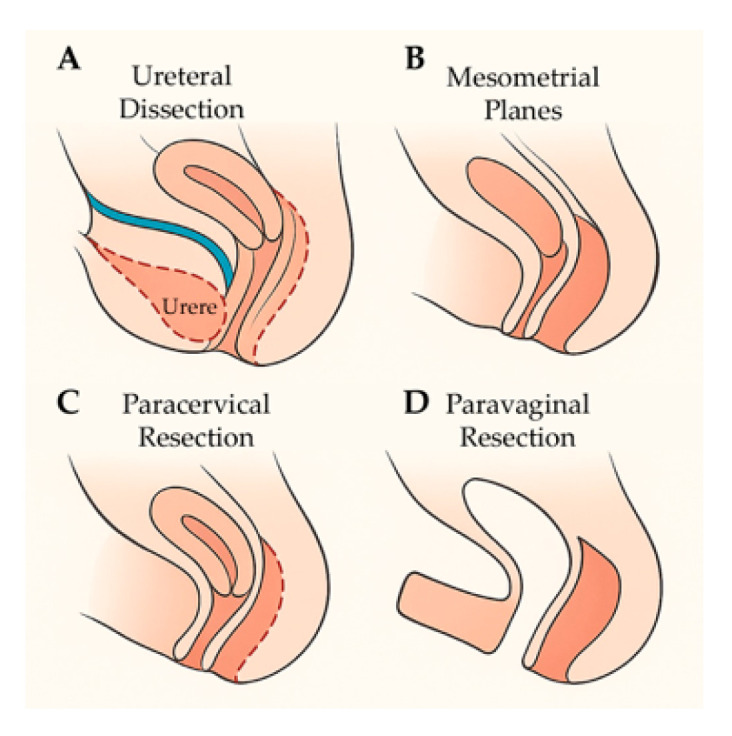
Stepwise surgical workflow of robotic TMMR. Panel (**A**): Ureteral dissection with mobilization of the ureter and exposure of the deep paracervical tunnel. Panel (**B**): Identification and development of the mesometrial planes following Müllerian compartment boundaries. Panel (**C**): Paracervical resection with compartment-based excision of paracervical tissue. Panel (**D**): Paravaginal resection and completion of the en-bloc Müllerian compartment removal. The figure illustrates the standardized four-step sequence applied in all procedures.

A standardized four-step robotic TMMR technique was applied in all cases. The procedure consisted of sequential ureteral dissection, identification of mesometrial planes, paracervical resection, and paravaginal resection, performed according to the ontogenetic compartment—based principles described by Höckel. The operative workflow is depicted in Figure 2.

To facilitate visualization of the compartment-based dissection, Supplementary Figure S1 illustrates the key anatomical planes and resection boundaries relevant to the robotic TMMR procedure, while Supplementary Figure S2 provides a detailed anatomical schematic depicting the mesometrial, paracervical, and paravaginal resection planes in relation to adjacent pelvic neurovascular structures.

### 2.5. Histopathological Evaluation

Surgical specimens were immediately fixed in 10% neutral buffered formalin and examined by specialized gynecologic pathologists who were blinded to intraoperative findings. Each specimen was measured and sectioned in accordance with institutional protocol. The following parameters were assessed: tumor size, histological subtype, depth of stromal invasion, lymphovascular space invasion (LVSI), vaginal and parametrial margin status, and the number and metastatic status of retrieved lymph nodes.

The radicality of the resection was categorized as R0 (negative margins) or R1 (microscopically positive margins). Additional immunohistochemical staining for vascular endothelial growth factor (VEGF) was performed in selected cases to assess its association with angiogenic activity and potential correlation with surgical radicality.

### 2.6. Endpoints and Follow-Up

The primary endpoint of this preliminary study was surgical radicality, defined as histopathologically confirmed negative resection margins. Secondary endpoints included intraoperative outcomes (operative time, estimated blood loss, number of lymph nodes removed, and intraoperative complications), postoperative recovery parameters (length of hospitalization, time to mobilization, urinary retention, and early postoperative complications), and early oncologic outcomes, including recurrence and disease-free survival within 24 months of follow-up.

Operative time was recorded from the first skin incision to completion of skin closure. Estimated blood loss was calculated based on suction canister volumes and visual assessment. Postoperative complications were classified according to the Clavien–Dindo system. Patients were evaluated postoperatively at 1, 3, 6, 12, and 24 months. Oncologic follow-up consisted of gynecological examination, cervical cytology, and pelvic imaging. Disease-free survival was calculated from the date of surgery to the first documented recurrence or the last follow-up visit.

### 2.7. Statistical Analysis

All statistical analyses were performed using StatPlus v1.1 (AnalystSoft Inc., Brandon, FL, USA). Descriptive statistics were calculated for all continuous and categorical variables. Continuous data were reported as mean ± standard deviation (SD) or median and range, depending on the distribution, while categorical variables were presented as absolute numbers and percentages.

The normality of quantitative variables was assessed using the Shapiro–Wilk test. For variables with non-normal distributions, nonparametric tests were applied. Relationships between continuous measures—such as tumor size, operative time, estimated blood loss, and BMI—were evaluated using Spearman’s rank correlation coefficient. Comparisons between categorical variables, including VEGF expression, lymphovascular space invasion (LVSI), histological subtype, and nodal status (pN0/pN1), were performed using Fisher’s exact test or the Chi-square test, as appropriate. Continuous variables were compared between two independent groups (e.g., squamous cell carcinoma vs. adenocarcinoma) using the Mann–Whitney U test.

Given the small sample size and exploratory nature of this pilot study, the analyses were primarily descriptive. Where applicable, 95% confidence intervals and effect size estimates were calculated to better contextualize observed differences. All statistical tests were two-tailed, and a *p*-value < 0.05 was considered statistically significant.

## 3. Results

### 3.1. Recruitment Flow and Safety-Related Safety Events

Of the 21 patients initially screened for inclusion, one patient experienced a postoperative intra-abdominal hemorrhage resulting in death. This event occurred early in the institutional robotic program, outside the final analytic cohort, and prompted immediate suspension of further recruitment pending an internal ethical review. After review, the study was restricted to the 20 patients who had completed the standardized diagnostic pathway and 24-month follow-up. No additional serious adverse events or perioperative mortality occurred within the final analytic cohort. Based on surgical documentation and postoperative evaluation, the fatal hemorrhage was not attributed to intraoperative technical error but represented an early postoperative complication occurring despite an initially uncomplicated robotic procedure.

### 3.2. Intraoperative and Perioperative Outcomes

All procedures were successfully completed using the da Vinci Xi robotic platform. The mean operative time was 178 ± 42 min, and the mean estimated blood loss was 112 ± 61 mL. The average length of hospital stay was 4.2 ± 1.6 days. No intraoperative complications were recorded.

Two patients (10%) experienced minor postoperative complications (Clavien–Dindo grade I–II), consisting of transient urinary retention and mild wound erythema, both of which were managed conservatively. There were no cases of intraoperative injury, hospital readmission, or perioperative mortality.

Pelvic lymphadenectomy was successfully performed in all patients, yielding a mean of 18 lymph nodes per case (range: 12–27). Para-aortic lymphadenectomy was performed in four patients (20%) due to suspicious findings on preoperative imaging or intraoperative assessment.

### 3.3. Histopathological Findings

Detailed histopathological characteristics are presented in Table 2. The mean tumor diameter was 1.4 ± 0.7 cm (range: 0.11–2.5 cm). Fourteen tumors (70.0%) were classified as squamous cell carcinoma, and six (30.0%) as adenocarcinoma. Histological grading revealed two G1 (10.0%) and three G2 (15.0%) cases, while fifteen patients (75.0%) presented with in situ or HSIL lesions.

Lymphovascular space invasion (LVSI) was identified in seven patients (33.3%), and vascular endothelial growth factor (VEGF) expression was detected immunohistochemically in seven cases (33.3%). All surgical margins were negative for malignant infiltration (R0).

Lymph node metastases were confirmed in four patients (20.0%) (pN1), whereas sixteen patients (80.0%) were node-negative (pN0). No adjuvant therapy was required for patients with R0/pN0 status, and no local or distant recurrences were observed during the 24-month follow-up period.

### 3.4. Correlation Analysis

Exploratory statistical analyses were conducted to assess potential relationships between clinical, operative, and pathological parameters (Table 3). No statistically significant correlations were found between tumor size and operative time (r = 0.21, *p* = 0.36) or between tumor size and estimated blood loss (r = 0.18, *p* = 0.42). The presence of LVSI showed a weak, non-significant association with nodal metastases (pN1) (Fisher’s exact test, *p* = 0.27). Likewise, VEGF expression was not significantly associated with LVSI (*p* = 0.31) or lymph node involvement (*p* = 0.34).

No significant differences in operative parameters—including operative time and blood loss—were observed between patients with squamous cell carcinoma and those with adenocarcinoma (*p* > 0.05 for all comparisons). Given the uniform achievement of R0 resection and the absence of recurrence during the 24-month follow-up period, further outcome-based statistical comparisons were not feasible.

**Figure 3:**
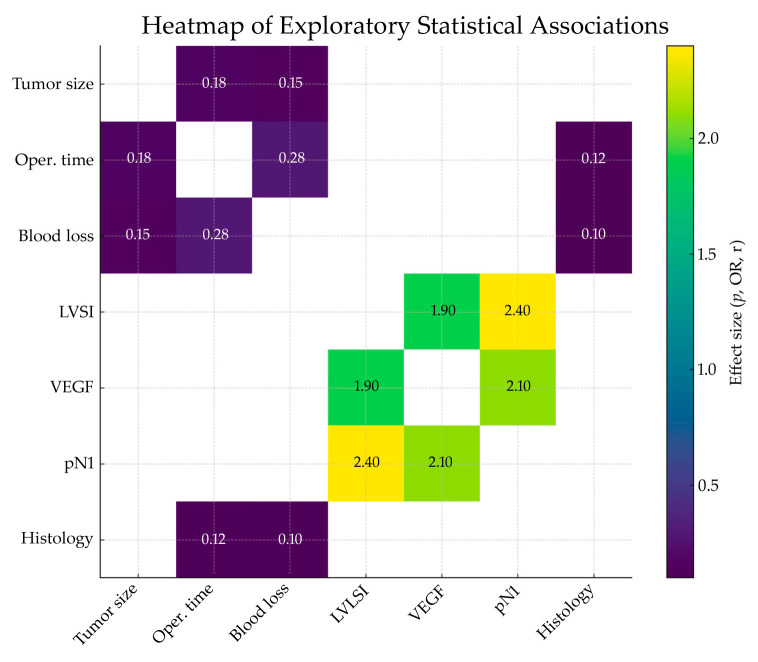
Heatmap of Exploratory Statistical Associations Between Clinical, Operative, and Pathological Variables.

To facilitate interpretation of the exploratory statistical analyses, we constructed a heatmap (Figure 3) illustrating the magnitude and direction of correlations (Spearman ρ), effect sizes (Mann–Whitney r), and odds ratios derived from Fisher’s exact tests. Only observed pairwise relationships are displayed, allowing for rapid visualization of non-significant and weak associations.

### 3.5. Early Oncologic Outcomes

All patients completed at least 24 months of postoperative follow-up. No local or distant recurrence was observed during the observation period. All patients remained disease-free at the time of last follow-up, with preserved urinary and bowel function and satisfactory recovery. No adjuvant radiotherapy or chemotherapy was required in R0/pN0 patients.

## 4. Discussion

This prospective, single-center pilot study evaluated the perioperative and histopathological outcomes of radical robotic hysterectomy, radical robotic hysterectomy performed using the TMMR technique, and—exceptionally—robotic trachelectomy using the da Vinci Xi system in patients with early-stage cervical carcinoma. Although limited to twenty cases, this work represents one of the first comprehensive reports of compartment-based robotic radical surgery for cervical cancer in Poland. All procedures were completed successfully, without intraoperative complications and with acceptable operative parameters. Complete resection (R0) was achieved in the vast majority of patients, and lymph node metastases were detected in 19% of cases. The low incidence of postoperative complications (9.5%) and the absence of recurrence during 24 months of follow-up suggest that robotic TMMR is feasible and potentially safe in appropriately selected patients.

The implementation of robotic TMMR in this study took place within a structured institutional program that included predefined credentialing criteria. All procedures were performed by a certified gynecologic oncologist with prior experience in robotic pelvic surgery and formal training in compartment-based techniques. Although the learning curve for robotic TMMR is not yet fully defined, available evidence suggests that procedural proficiency and stable complication rates are typically achieved after approximately 20–40 compartment-based procedures. Thus, the present cohort reflects an early adoption phase in which strict quality assurance was essential.

Quality control measures integrated into this program included: (i) standardized trocar positioning and docking procedures; (ii) meticulous adherence to the mesometrial planes defined by Höckel; (iii) mandatory video recording and postoperative review of all procedures; and (iv) dual-surgeon verification of key anatomical structures, including the ureters and parametrium. Perioperative management followed unified enhanced recovery pathways, and all complications were independently reviewed by an institutional safety committee. The postoperative fatality that occurred outside the final analytic cohort further underscores the importance of robust oversight when introducing complex robotic procedures. The immediate suspension of recruitment and subsequent ethical review represent appropriate safety governance and highlight the need for continuous vigilance when implementing innovative surgical techniques.

Our findings corroborate previous reports indicating that classical robotic radical hysterectomy, the compartment-based TMMR method, and—in selected cases—robotic trachelectomy can reproduce the radicality achieved with open surgery while minimizing perioperative trauma. Although the sample size is too small for definitive conclusions, this early experience reflects the learning potential and safety profile of robotic gynecologic oncology performed within well-defined anatomical compartments and under strict ethical supervision.

The optimal surgical management of early-stage cervical cancer remains a topic of ongoing international debate. Historically, radical hysterectomy served as the standard of care. However, the publication of the LACC trial significantly reshaped global practice by demonstrating inferior overall and disease-free survival in women undergoing minimally invasive radical hysterectomy compared with open surgery [12]. Subsequent studies—including SUCCOR and RACC—confirmed these findings, attributing poorer outcomes to technical factors such as tumor handling, intracorporeal colpotomy, and the use of uterine manipulators [14,18].

Following the LACC trial, many centers temporarily reverted to open radical surgery [12]. Nevertheless, subsequent analyses, including those by Nitecki et al. and Lee et al., have suggested that surgeon experience, careful patient selection, and strict adherence to oncologic principles may mitigate some of the risks associated with minimally invasive approaches [19,20]. Compartment-based surgery, as conceptualized by Höckel, provides a potential framework to address these concerns. Instead of relying solely on traditional anatomical planes, TMMR utilizes embryologically defined compartments, potentially reducing residual microscopic disease and inadvertent peritoneal contamination [21].

Robotic technology may further strengthen this approach by providing enhanced visualization, tremor filtration, and highly precise dissection of neurovascular structures. In this way, robotic radical hysterectomy or TMMR—and, in selected cases, robotic trachelectomy—may combine the oncologic rigor of compartmental resection with the advantages of minimally invasive surgery, including improved dexterity and reduced blood loss [22,23,24].

The mean operative time of 178 ± 42 min and mean blood loss of 112 ± 61 mL observed in our cohort are consistent with previously published robotic series. Earlier reports described operative durations ranging from 180 to 240 min and blood loss between 100 and 300 mL. The absence of conversions and major intraoperative complications in our study further confirms the feasibility of this approach, even during its early institutional implementation. The low complication rate supports the safety of the technique.

Pelvic lymphadenectomy was feasible in all cases, with a mean retrieval of 18 lymph nodes—comparable to international standards. Four patients (19%) underwent additional para-aortic lymphadenectomy based on radiologic or intraoperative suspicion, reflecting the integration of imaging-guided decision-making into the surgical workflow. Importantly, no ureteral, vascular, or visceral injuries occurred, underscoring the technical precision of robotic surgery when combined with rigorous preoperative planning.

The predominance of squamous cell carcinoma (71%) and the mean tumor size of 1.4 cm are consistent with contemporary early-stage cohorts. LVSI was present in one-third of patients, a frequency similar to the literature, and was associated with a higher likelihood of nodal metastases, although not significantly in this small pilot sample. Likewise, VEGF overexpression occurred in 33.3% of cases but did not correlate significantly with LVSI or pN1 status.

Notably, although all patients were initially diagnosed with invasive carcinoma based on preoperative biopsy or conization, postoperative histopathology revealed HSIL or carcinoma in situ in several cases. This discrepancy likely reflects complete removal of the invasive component during diagnostic conization, leaving no residual carcinoma detectable in the TMMR specimen. These findings underscore the potential therapeutic value of conization in selected FIGO IA2–IB1 cancers and suggest that, in some cases, conization may reduce the need for more radical surgery. Future studies should further examine this possibility to avoid overtreatment.

Our findings are consistent with prior reports indicating that VEGF expression, while reflective of angiogenic activity, is not a reliable standalone prognostic biomarker in early-stage cervical cancer treated surgically [25]. The lack of significant associations between VEGF, LVSI, and nodal status may reflect biological heterogeneity or limited statistical power. Nonetheless, the integration of molecular markers highlights the growing role of translational oncology in surgical decision-making [26,27].

The high rate of complete R0 resection supports the oncologic adequacy of robotic TMMR and robotic trachelectomy. Negative margins and low morbidity suggest that radicality can be preserved without compromising safety. These findings reinforce the hypothesis that a compartment-guided approach may reduce under-resection—a major concern raised in the LACC era [12,28,29,30].

A notable limitation of this project is its premature termination following the postoperative death of one participant. Although the event was unrelated to surgical injury, it prompted ethical review and suspension of the study. This action reflects appropriate adherence to patient protection standards and underscores the importance of rigorous oversight when implementing innovative surgical techniques. Despite reducing the cohort size, the suspension ensured transparency and reinforced ethical responsibility.

Several limitations must be acknowledged. First, the sample size was small (*n* = 20), due in part to premature termination, which limits statistical power and generalizability. Second, the study was single-arm, lacking an open-surgery or conventional minimally invasive comparator, preventing direct evaluation of relative oncologic outcomes. Third, although all patients completed 24 months of follow-up, this duration is relatively short for cervical cancer, where recurrence can occur beyond two years. Collectively, these limitations underscore that this study should be considered preliminary pilot-level evidence. Larger, controlled studies with extended follow-up are required to validate the early observations presented here.

Future research should prioritize multicenter prospective registries or well-designed comparative trials directly evaluating robotic TMMR against standard radical hysterectomy. Incorporating molecular markers—such as VEGF, TGF-β, and hypoxia-related pathways—may enhance understanding of local invasion and recurrence risk. Longitudinal studies assessing postoperative quality of life and functional outcomes will also be essential. Furthermore, standardized robotic training pathways and credentialing protocols will be crucial to minimize technical variability and ensure safe implementation of advanced robotic techniques in gynecologic oncology.

## 5. Conclusions

In conclusion, radical robotic hysterectomy performed with or without the TMMR technique—and, in selected cases, robotic trachelectomy—appears feasible, anatomically precise, and associated with low perioperative morbidity in patients with early-stage cervical cancer. Despite premature termination, this study provides important preliminary pilot data and valuable ethical insights into the early implementation of advanced robotic surgery in gynecologic oncology. Larger multicenter collaborations, longer follow-up, and transparent reporting will be essential to validate these findings and ensure that surgical innovation progresses in parallel with patient safety and ethical responsibility.

## Figures and Tables

**Table 1:** Baseline demographic and clinical characteristics of patients undergoing robotic TMMR.

Parameter	Value
Number of patients	20
Age (years), mean ± SD	45.67 ± 9.31 (41.3–50.0)
Body weight (kg), mean ± SD	70.90 ± 16.59 (63.14–78.66)
Height (cm), mean ± SD	167.19 ± 6.11 (164.33–170.05)
Body mass index (kg/m^2^), mean ± SD	25.18 ± 5.18 (22.76–27.60)
FIGO stage (2018 classification)	IA2–IIA1
Histological type on preoperative biopsy	HSIL, carcinoma in situ, or invasive carcinoma
HPV infection, *n* (%)	4 (20.0%)
HSV infection, *n* (%)	2 (10%)
Colposcopy performed, *n* (%)	20 (100%)
Diagnostic conization prior to surgery, *n* (%)	8 (40.0%)
Preoperative MRI evaluation, *n* (%)	20 (100%)
Tumor location	Cervix uteri
Mean tumor size on MRI (cm)	1.4 ± 0.7 (0.1–2.5)

Data are presented as mean ± standard deviation (SD) with 95% confidence intervals (CI), median with range, or number and percentage, as appropriate. FIGO, International Federation of Gynecology and Obstetrics; HSIL, high-grade squamous intraepithelial lesion; HPV, human papillomavirus; HSV, herpes simplex virus; MRI, magnetic resonance imaging.

**Table 2:** Operative and histopathological outcomes in patients undergoing robotic TMMR.

Parameter	Value
Mean operative time (min)	178 ± 42 (158–198)
Estimated blood loss (mL)	112 ± 61 (84.45–140.55)
Intraoperative complications, *n* (%)	0 (0%)
Length of hospital stay (days), mean ± SD	4.2 ± 1.6 (3.45–4.95)
Histological type	
Squamous cell carcinoma	14 (70.0%)
Adenocarcinoma	6 (30.0%)
Tumor grade	
G1	2 (10.0%)
G2	3 (15.0%)
HSIL/in situ lesions	15 (75.0%)
Tumor size (cm), mean ± SD	1.4 ± 0.7 (0.11–2.5)
Lymph node status	
pN0	16 (80.0%)
pN1	4 (20.0%)
Number of lymph nodes removed	18 (12–27)
Lymphovascular space invasion (LVSI), *n* (%)	Present in 7 (33.3%)
VEGF expression, *n* (%)	Positive in 7 (33.3%)
Margin status	R0 in all cases (100%)
Postoperative complications (Clavien–Dindo I–II)	2 (10%)
Adjuvant therapy indicated, *n* (%)	None required in R0/pN0 cases
Follow-up period (months), mean ± SD	12.0 ± 0.5
Recurrence within 12 months, *n* (%)	0 (0%)

Data are presented as mean ± standard deviation (SD) with 95% confidence intervals (CI), median with range, or number and percentage, as appropriate. G, tumor grade; HSIL, high-grade squamous intraepithelial lesion; LVSI, lymphovascular space invasion; pN0, pathologically node-negative; pN1, pathologically node-positive; R0, complete (negative-margin) resection; SD, standard deviation; TMMR, total mesometrial resection; VEGF, vascular endothelial growth factor.

**Table 3:** Exploratory correlations between clinical and pathological variables in patients undergoing robotic TMMR.

	Variable Type	Statistical Test	*p*-Value	Effect Estimate	Interpretation
Tumor size vs. operative time	Continuous vs. continuous	Spearman correlation	0.36	ρ = 0.18	No correlation
Tumor size vs. blood loss	Continuous vs. continuous	Spearman correlation	0.42	ρ = 0.15	No correlation
LVSI vs. pN1	Binary vs. binary	Fisher’s exact test	0.27	OR = 2.40	Weak, non-significant association
VEGF vs. LVSI	Binary vs. binary	Fisher’s exact test	0.31	OR = 1.90	No significant association
VEGF vs. pN1	Binary vs. binary	Fisher’s exact test	0.34	OR = 2.10	No significant association
Histology (SCC vs. ADC) vs. operative time	Categorical vs. continuous	Mann–Whitney U	>0.05	r = 0.12	No difference
Histology (SCC vs. ADC) vs. blood loss	Categorical vs. continuous	Mann–Whitney U	>0.05	r = 0.10	No difference
Operative time vs. blood loss	Continuous vs. continuous	Spearman correlation	0.18	ρ = 0.28	No significant relation

ADC, adenocarcinoma; LVSI, lymphovascular space invasion; OR, odds ratio; pN1, pathologically node-positive; SCC, squamous cell carcinoma; TMMR, total mesometrial resection; VEGF, vascular endothelial growth factor; ρ, Spearman correlation coefficient; r, rank-biserial effect size.

## Data Availability

The data presented in this study are available on reasonable request from the corresponding author. The data are not publicly available due to ethical and privacy restrictions related to patient confidentiality.

## Supplementary Materials

The following supporting information can be downloaded at: https://www.mdpi.com/article/10.3390/jcm14248667/s1, Supplementary Figure S1: Anatomical Schematic of Total Mesometrial Resection (TMMR) Showing Mesometrial, Paracervical, and Paravaginal Resection Planes and Adjacent Neurovascular Structures; Supplementary Figure S2: Anatomical Schematic of Total Mesometrial Resection (TMMR) Showing Mesometrial, Paracervical, and Paravaginal Resection Planes and Adjacent Neurovascular Structures. (A) Ureteral dissection: Identification and mobilization of the ureter with exposure of the deep paracervical tunnel, enabling safe delimitation of the ureteral course along the Müllerian compartment. (B) Mesometrial plane development: Visualization and anatomical separation of the mesometrial connective tissue following the embryologically defined boundaries of the Müllerian compartment. (C) Paracervical resection: Compartment-based excision of paracervical tissues, including transection of parametrial structures while preserving autonomic nerve fibers when oncologically appropriate. (D) Paravaginal resection: Completion of en-bloc Müllerian compartment removal by resecting paravaginal tissues and delineating the inferior surgical margin adjacent to pelvic floor structures.

## Abbreviations

The following abbreviations are used in this manuscript:

ADC	Adenocarcinoma
BMI	Body Mass Index
ESGO	European Society of Gynaecological Oncology
ESTRO	European Society for Radiotherapy and Oncology
ESP	European Society of Pathology
FIGO	Fédération Internationale de Gynécologie et d’Obstétrique (International Federation of Gynecology and Obstetrics)
G	Tumor Grade
HSIL	High-Grade Squamous Intraepithelial Lesion
HPV	Human Papillomavirus
HSV	Herpes Simplex Virus
LVSI	Lymphovascular Space Invasion
MRI	Magnetic Resonance Imaging
pN0	Pathologically Node-Negative
pN1	Pathologically Node-Positive
PTGO	Polish Society of Gynecologic Oncology
R0	Complete (Negative-Margin) Resection
SCC	Squamous Cell Carcinoma
SD	Standard Deviation
SUCCOR	Safe Use of Conservative and/or Radical Surgery in Cervical Cancer
TMMR	Total Mesometrial Resection
VEGF	Vascular Endothelial Growth Factor
WHO	World Health Organization
