# Supplementary material for: Outcomes of Cervical Cancer Treatment Using Total Mesometrial Resection (TMMR) Performed with the Robotic System—A Preliminary Report
Source: J Clin Med. 2025 Dec 7;14(24):8667. doi: 10.3390/jcm14248667 (PMC12733901; doi:10.3390/jcm14248667)
Supplement: Supplementary file 1 [file jcm-14-08667-s001.zip › jcm-3963290-supplementary.pdf]

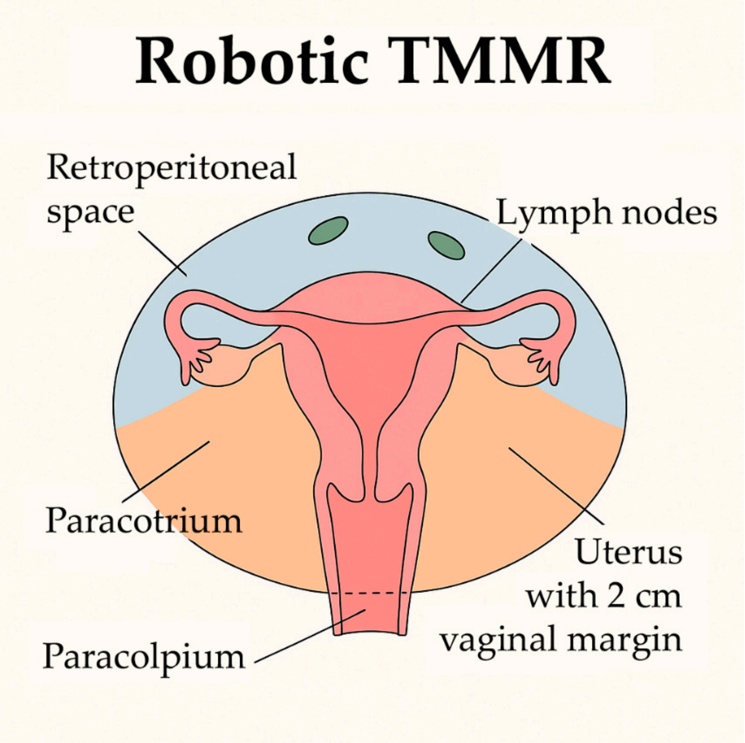

Supplementary Figure S1. Anatomical Schematic of Total Mesometrial Resection (TMMR) Showing Mesometrial, Paracervical, and Paravaginal Resection Planes and Adjacent Neurovascular Structures.

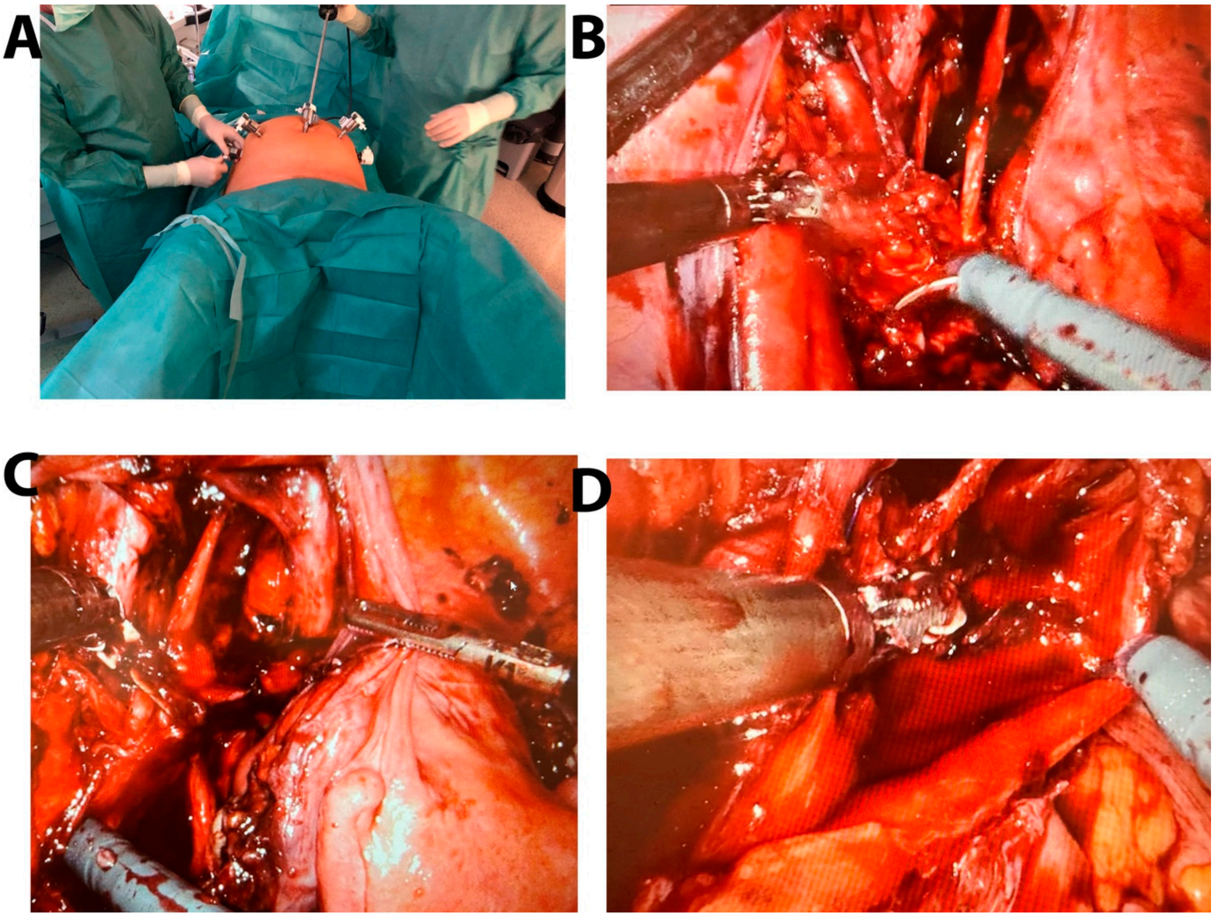

Supplementary Figure S2. Anatomical Schematic of Total Mesometrial Resection (TMMR) Showing Mesometrial, Paracervical, and Paravaginal Resection Planes and Adjacent Neurovascular Structures.
(A) Ureteral dissection: Identification and mobilization of the ureter with exposure of the deep paracervical tunnel, enabling safe delimitation of the ureteral course along the Müllerian compartment.
(B) Mesometrial plane development: Visualization and anatomical separation of the mesometrial connective tissue following the embryologically defined boundaries of the Müllerian compartment.
(C) Paracervical resection: Compartment-based excision of paracervical tissues, including transection of parametrial structures while preserving autonomic nerve fibers when oncologically appropriate.
(D) Paravaginal resection: Completion of en-bloc Müllerian compartment removal by resecting paravaginal tissues and delineating the inferior surgical margin adjacent to pelvic floor structures.
